# Association of Polymorphisms in Vitamin D-Metabolizing Enzymes *DHCR7* and *CYP2R1* with Cancer Susceptibility: A Systematic Review and Meta-Analysis

**DOI:** 10.1155/2021/6615001

**Published:** 2021-05-22

**Authors:** Jing Wen, Jia Li, Xinyuan Liang, Aiping Wang

**Affiliations:** ^1^Physical Examination Center, The First Hospital of China Medical University, Shenyang, Liaoning 110001, China; ^2^Department of Nursing, The First Hospital of China Medical University, Shenyang, Liaoning 110001, China

## Abstract

The deficiency of vitamin D has been reported to be relevant to cancer risk. *DHCR7* and *CYP2R1* are crucial components of vitamin D-metabolizing enzymes. Thus, accumulating researchers are concerned with the correlation between polymorphisms of *DHCR7* and *CYP2R1* genes and cancer susceptibility. Nevertheless, the conclusions of literatures are inconsistent. We conducted an integrated review for the correlation of *DHCR7* and *CYP2R1* SNPs with cancer susceptibility. In the meanwhile, a meta-analysis was performed using accessible data to clarify the association between *DHCR7* and *CYP2R1* SNPs and overall cancer risk. Literatures which meet the rigid inclusion and exclusion criteria were involved. The association of each SNP with cancer risk was calculated by odds ratios (ORs). 12 case-control designed studies covering 23780 cases and 27307 controls were ultimately evolved in the present meta-analysis of five SNPs (*DHCR7* rs12785878 and rs1790349 SNP; *CYP2R1* rs10741657, rs12794714, and rs2060793 SNP). We found that *DHCR7* rs12785878 SNP was significantly related to cancer risk in the whole population, Caucasian subgroup, and hospital-based (HB) subgroup. *DHCR7* rs1790349 SNP was analyzed to increase cancer risk in Caucasians. Moreover, *CYP2R1* rs12794714-A allele had correlation with a lower risk of colorectal cancer. Our findings indicated that rs12785878, rs1790349, and rs12794714 SNPs might potentially be biomarkers for cancer susceptibility.

## 1. Introduction

Vitamin D, also regarded as 1,25-dihydroxyvitamin D3, is a pivotal steroid prohormone which has a significant role to play in musculoskeletal health [1]. Additionally, compelling evidence reveals the roles of vitamin D on extraskeletal diseases, such as infectious disease [2], cardiovascular disease [3], autoimmune disease [4], neurodegeneration [5], and cancer [6]. Deficiency of vitamin D has been reported to be relevant to oral squamous cell carcinoma [7], breast cancer [8], colorectal cancer [9], prostate cancer [10], pancreas cancer [11], thyroid cancer [12], hepatocellular carcinoma [13], and ovarian cancer [14]. Furthermore, vitamin D supplementation may decrease the death of cancer by 16% [15].

There has an individual variability in serum vitamin D stores which cannot be explained alone by age, sunlight exposure, body mass index, or dietary intake [12]. Studies have demonstrated that vitamin D level is highly heritable [16]. Genetic and epigenetic factors can impact several crucial steps along the metabolic pathway of vitamin D. Genes who directly participate in the vitamin D pathway gene are *DHCR7*, *CYP2R1*, *VDR*, *CYP24A1*, *CYP27B1*, and so on, and the aberrant expressions of them have been demonstrated to be associated with vitamin D concentrations and cancer [17–21]. Genome-wide association studies (GWAS) have detected the correlations of 25-hydroxyvitamin D concentrations with single nucleotide polymorphisms (SNPs) on genes that participated in the vitamin D metabolic pathway [1, 16].

*DHCR7*, located on chromosome 11q13.4, encodes ultimate enzyme 7-dehydrocholesterol reductase which catalyzes the conversion of the vitamin D3 precursor (7-dehydrocholesterol) to cholesterol, instead of vitamin D3 [22]. Cytochrome P450 family 2 subfamily R member 1 (*CYP2R1*, on chromosome 11p15.2) encodes vitamin D 25-hydroxylase which catalyzes the initial hydroxylation reaction of vitamin D synthesis, converting vitamin D to 25-hydroxyvitamin D [9]. Increasing correlational studies were concerned with *DHCR7* and *CYP2R1* polymorphisms and susceptibility to cancer. Some studies confirmed the associations, whereas others remained skeptical or denied their correlations. The aim of the present study was to explore whether the *DHCR7* or *CYP2R1* SNPs are related to cancer risk.

We comprehensively reviewed the eligible studies and analyzed all available data. Our aim is to explore the association of *DHCR7* and *CYP2R1* SNPs with cancer risk, supplying clues to researchers for screening novel cancer biomarkers.

## 2. Materials and Methods

### 2.1. Retrieval Strategy

Two investigators (J.W. and J.L.), respectively, carried out a comprehensive literature retrieval in PubMed and Web of Science database up to February 2020, by using the following query terms: “*CYP2R1*/cytochrome P450 family 2 subfamily R member 1/*DHCR7*/7-dehydrocholesterol reductase”, “polymorphism/SNP/variant/variation”, and “cancer/carcinoma/neoplasm/tumor/”. All enrolled articles must satisfy inclusion standards: (1) case-control or nested case-control designed study; (2) in regard to the association of *DHCR7* and *CYP2R1* SNPs with predisposition to cancer. Meanwhile, publications meeting the following exclusion standards were removed: (1) letters or reviews; (2) repeated records; (3) irrelevant to *DHCR7* and *CYP2R1* SNPs or carcinoma; (4) without any available genotype distribution data.

### 2.2. Data Extraction

Data was collected by two investigators (J.W. and J.L.) independently and came to a consensus regarding all items. Essential characteristics extracted from each qualified publication comprised first author, year of publication, ethnicity, sample size, type of carcinoma, gene, SNPs, genotype distribution frequency of case and control groups, control group source (hospital-based (HB) or population-based (PB)), Hardy-Weinberg equilibrium (HWE), adjustment factors, and genotyping method. When multiple studies were conducted in one article, data were collected individually.

### 2.3. Methodology Quality Assessment

Two authors (J.W. and X.L.) scored the quality of each enrolled publication independently, based on a scoring scheme mentioned in prior literature [23, 24]. Six evaluation items were involved in the scoring scheme: representativeness of cases, control source, ascertainment of carcinomas, sample size, HWE in the control group, and quality assurance of genotyping methods. The quality assessment scores ranged from 0 to 10. Study with no less than 5 quality scores was recognized as an eligible study which could be enrolled in subsequent analysis.

### 2.4. False-Positive Report Probability

False-positive report probability (FPRP) was computed to estimate whether our study findings are “noteworthy.” Initially, we computed the statistic power of the test based on the sample size, ORs, and *P* values by using NCSS-PASS software (USA, version 11.0.7). Then, we drew the FPRP values from a calculation formula which had been reported in earlier researches, and FPRP < 0.5 was regarded as a noteworthy finding [25].

### 2.5. Statistical Analysis

The chi-square test (*χ*^2^ test) was conducted to compute the HWE for genotype frequency distribution of *CYP2R1* and *DHCR7* polymorphisms in controls. The correlation of each *CYP2R1* and *DHCR7* polymorphism with carcinoma risk was computed by odds ratio (OR) with its 95% confidence interval (95% CI). Cochran's *χ*^2^-based *Q* test was adopted to estimate the heterogeneity of interstudy (significance set as *P* < 0.10, *I*^2^ > 50%). We pooled the results by means of a fixed-effects model when no interstudy heterogeneity arose; the random-effects model was adopted otherwise. Besides, the recessive and dominant genetic models were, respectively, considered as variant homozygote vs. heterozygote/wild homozygote, and heterozygote/variant homozygote vs. wild homozygote. Publication bias was estimated using the rank correlation test (Begg's test) and linear regression methods (Egger's test). Sensitivity analysis was calculated to show whether the merged findings were steady enough after removing those outlying studies. All the mentioned statistical analyses were calculated by STATA software (STATA Corp., College Station, TX, USA, version 11.0). All *P* values were for two-tailed tests, and less than 0.05 was regarded as statistically significant.

## 3. Results

### 3.1. Features of Eligible Studies and Analyzed SNPs

**Figure 1:**
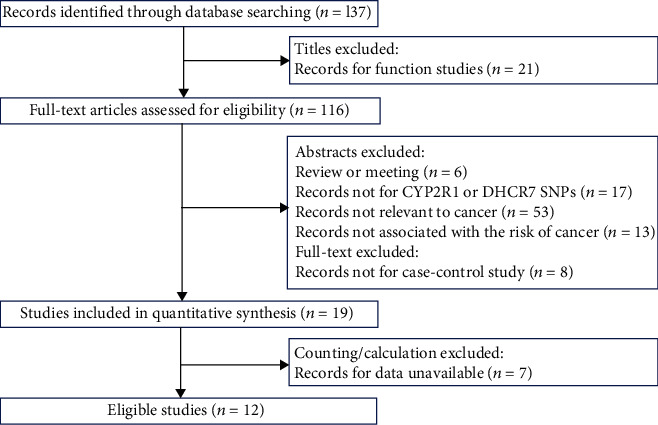
The flow chart of identification for studies included in the meta-analysis based on PRISMA guidelines.

Totally 137 publications were gathered through database retrieval after removing duplicate hits. 125 articles were removed after browsing titles and abstracts: 21 were functional studies; 6 were review or meeting; 8 were not case-control studies; 17 were not related to *DHCR7* or *CYP2R1* SNPs; 53 were not concerned with carcinoma; and 13 were not correlated with carcinoma risk. Therefore, 19 studies are ought to be involved in the present analysis. Nevertheless, 7 publications lost original data, 5 of which were genome-wide association studies. And we were not able to contact with authors. Thus, 12 case-control designed studies were finally evolved in the present meta-analysis, covering 23780 cases and 27307 controls, which is shown in Figure 1. The features of these eligible studies which met the quality assessment criterion are listed in Table 1.

Six polymorphisms were able to be involved in our systematic review, including rs10741657 G/A, rs12794714 G/A, rs2060793 G/A, rs3829251 G/A, rs12785878 T/G, and rs1790349 A/G. The frequency distribution of *DHCR7* and *CYP2R1* SNPs genotype is shown in Table 2. Six records, however, were removed from quantitative synthesis owing to the insufficient study number for some loci or being not conformed to HWE (*P*^HWE^ < 0.05). Consequently, five SNPs were covered in the eventual meta-analysis. For *DHCR7*, the analyzed SNPs were rs12785878 T/G and rs1790349 A/G; for *CYP2R1*, the analyzed SNPs were rs10741657 G/A, rs12794714 G/A, and rs2060793 G/A.

### 3.2. Quantitative Data Synthesis of Five SNPs in *DHCR7* and *CYP2R1* Genes

#### 3.2.1. Two Polymorphisms in DHCR7 Gene

Five eligible studies were collected to evaluate the relationships between *DHCR7* SNPs and risk of carcinoma, on the basis of entire population. The rs12785878 T/G SNP was illustrated to be associated with incremental cancer risk. The correlation of rs12785878 T/G SNP was discovered under the heterozygote genotype model (TG vs. TT: OR (95%CI) = 1.168 (1.027-1.328), *P* = 0.018, Table 3). The relationship between rs1790349 A/G SNP and carcinoma risk was not found in the initial analysis.

In stratified analyses, rs12785878 T/G SNP was quantitatively analyzed in “ethnicity,” “type of carcinoma,” and “source of control group” subgroups, and the rs1790349 A/G SNP was analyzed in the “ethnicity” subgroup. For rs12785878 T/G SNP, correlations calculated under the heterozygote genotype model (TG vs. TT) were observed in “Caucasian population” and “PB” subgroups (Caucasian: OR (95%CI) = 1.178 (1.021-1.358), *P* = 0.024; PB: OR (95%CI) = 1.193 (1.028-1.385), *P* = 0.020, Table 3). For rs1790349 A/G SNP, association was only manifested in the “Caucasian population” subgroup (AG vs. AA: OR (95%CI) = 1.201 (1.008-1.431), *P* = 0.040, Table 3).

#### 3.2.2. Three Polymorphisms in CYP2R1 Gene

Nine eligible publications were involved to estimate the association intensity of *CYP2R1* polymorphisms and overall carcinoma risk. Nevertheless, none of these SNPs manifest significant correlations with risk of carcinoma in any genetic models.

Then, stratified analyses of rs10741657 G/A and rs12794714 G/A SNPs were conducted based on “ethnicity,” “type of carcinoma,” and “source of control group,” on account of the presence of between-study heterogeneity. For rs12794714 G/A SNP, its allelic models had correlation with a decreased genetic predisposition to colorectal cancer (A vs. G: OR (95%CI) = 0.866 (0.753-0.997), *P* = 0.046, Table 3). Correlations could not be elucidated among any of the stratified analyses of rs10741657 G/A SNP.

### 3.3. Sensitivity Analysis

Sensitivity analysis was adopted to assess the impact of each study on summarized findings, by means of calculating the OR (95% CI) before and after deleting each article from the pooled analysis. For rs12785878 T/G SNP, it made no sense after the removal of two articles (Isabel S. Carvalho 2019, Tess V. Clendenen 2015) individually (Supplementary Table S1).

### 3.4. Publication Bias

Potential publication bias was evaluated for all covered publications by means of two test methods mentioned above. The publication bias was found in rs12794714 G/A SNP under the recessive model, for *P* < 0.1 in both tests, which might be because of the deficient publications with negative results or the defective methodological design for small-scale studies (Table 4).

### 3.5. FPRP Analyses

Eventually, we assessed the FPRP for our significant findings. For studies of uncommon neoplasm or common tumors with small sample size, the FRPR value less than 0.5 would make a massive improvement over previous practice, based on the professional guide of FPRP calculation. Since the present study is the first meta-analysis to estimate the association between DHCR7 and CYP2R1 SNPs and cancer risk, we consider 0.5 as the FPRP threshold. The FPRP values of rs12785878 SNP (prior probability 0.25/0.1) were less than 0.5, and FPRP values of rs1790349 and rs12794714 SNPs were also less than 0.5 (prior probability 0.25), suggesting these significant associations are deserving of attention (Table 5).

## 4. Discussion

In the present article, a comprehensive review was performed for the correlation of SNPs in *DHCR7* and *CYP2R1* genes with overall cancer risk. And a meta-analysis was conducted for five prevalent SNPs (*DHCR7*: rs12785878 T/G and rs1790349 A/G; *CYP2R1*: rs10741657 G/A, rs12794714 G/A, and rs2060793 G/A) for the first time. Our findings showed that rs12785878, rs1790349, and rs12794714 SNPs were related to cancer susceptibility in the whole population or in some subgroups, which means they might participate in cancerogenesis. No associations were discovered in other polymorphisms.

### 4.1. Polymorphisms in *DHCR7*

*DHCR7* encodes an enzyme 7-dehydrocholesterol reductase which converts 7-dehydrocholesterol into cholesterol. This enzyme is a critical regulatory switch between vitamin D3 and cholesterol, for both biosynthesis processes require 7-dehydrocholesterol as substrate [26]. Moreover, *DHCR7* has been assumed to be a correlated gene for vitamin D concentration and carcinoma risk [1].

Regarding rs12785878 T/G, it has been illustrated to be a 25(OH) D concentration-related SNP [1]. We found significant correlations between rs12785878 SNP and cancer susceptibility in the whole population, Caucasian subgroup, and population-based subgroup. rs12785878 SNP is located 8000 bases upstream from 5 prime UTR region of *DHCR7*, and it is still unclear whether it has an impact on gene expression or has a linkage disequilibrium with some other functional SNPs. The present meta-analysis of rs12785878 SNP encompasses 5 case-control studies. Only one of the five studies, however, was in accordance with our consequence. For the rs1790349 A/G SNP, it was computed to be associated with cancer risk in the Caucasian subgroup under heterozygote genotype. The rs1790349 SNP is located in the intergenic region near *DHCR7* and has also been identified to be a 25(OH) D concentration-associated SNP in genome-wide association study [16, 27, 28]. Our analysis of rs1790349 SNP involves only 2 case-control studies, so further expansion of sample volume is needed.

### 4.2. Polymorphisms in *CYP2R1*

*CYP2R1*, as a vital important 25-hydroxylase, metabolizes vitamin D to 25(OH) D in the liver [29]. The genetic variations in *CYP2R1* were correlated with the impaired activity of 25-hydroxylases, which influence the serum 25(OH) D level [30]. Association of serum 25(OH) D level with cancer susceptibility has been revealed in breast cancer [20], gastric cancer [31], thyroid cancer [32], prostate cancer [33], colorectal cancer [34], and so on. Thus, accumulating researchers were concerned with the correlation between *CYP2R1* SNPs and cancer susceptibility.

For rs12794714 (G/A) SNP, we analyzed a significant relationship between A allele-rs12794714 SNP and decreased risk of colorectal cancer (CRC). Located in exon 1 region of *CYP2R1*, rs12794714 G/A SNP may function as an exon splicing enhancer (ESE)/exon splicing silencer (ESS) to impact gene expression, whereas it is a synonymous variant (https://snpinfo.niehs.nih.gov/). The A allele-rs12794714 SNP has been illustrated to be associated with higher serum 25-hydroxyviatamin D concentrations [16]; thus, it may reduce the cancer risk. Thus far, the protective effect of rs12794714 has only been demonstrated in CRC. Further studies remain desired concerning rs12794714 and cancer.

### 4.3. Limitations and Conclusions

It ought to be mentioned that the present study has several limitations. First and foremost, association studies of *DHCR7* and *CYP2R1* polymorphisms with cancer predisposition remain limited. Further researches are demanded for updated meta-analyses. Moreover, several items without accessible original records were removed from ultimate analysis, which might cause publication bias.

Overall, we comprehensively assessed the correlation of *DHCR7* and *CYP2R1* SNPs with carcinoma risk. Additionally, a meta-analysis was conducted based on all accessible data for five polymorphisms. The consequence demonstrated that 3 (re12794714, rs12785878, and rs1790349) of the 5 SNPs were associated with cancer risk in whole population or in some subgroups, indicating that they might be feasible biomarkers for cancer susceptibility.

## Figures and Tables

**Table 1:** Characteristics of eligible studies.

No.	First author	Year	Ethnicity	Sample size		Source of control groups	Genotyping method	Adjusted factors	Citation
				Case	Control				
1	Isabel S. Carvalho	2019	Caucasian (Portugal)	500	500	PB	PCR-RFLP	Age, sex	[12]
2	Prajjalendra Barooah	2019	Caucasian (Indian)	60	102	HB	PCR-RFLP	Age, sex	[13]
3	Jianzhou Yang	2017	Asian (China)	565	557	PB	GenomeLab SNPstream	Age, sex	[35]
4	Alison M. Mondul	2015	Caucasian (European)	8618	9960	HB	TaqMan or genome-wide scans	Age	[36]
5	Tess V. Clendenen	2015	Caucasian (Swedish)	733	1432	PB	Illumina GoldenGate assay	Age, menopausal status	[37]
6	Fabio Pibiri	2014	African (African-American)	902	760	PB	Sequenom MassARRAY	Age, sex, ancestry	[38]
7	Touraj Mahmoudi	2014	Caucasian (Iranian)	290	354	HB	PCR-RFLP	Age, BMI, sex	[9]
8	Wei Wang	2014	Caucasian (Hispanic)	826	779	PB	Illumina GoldenGate assay	Age, BMI	
	Wei Wang	2014	Mixed (non-Hispanic)	224	130	PB	Illumina GoldenGate assay	Age, BMI	[39]
9	Christian M. Lange	2013	Asian (Japanese)	803	1253	HB	Competitive allele-specific TaqMan PCR	Sex	[40]
10	Alison M. Mondul	2013	Caucasian	9378	9986	PB	TaqMan	Age, ethnicity	[41]
11	Laura N. Anderson	2013	Caucasian (Canada)	628	1192	PB	MassARRAY	Age, sex	[11]
12	Marissa Penna-Martinez	2012	Caucasian (Germany)	253	302	PB	TaqMan	NM	[42]

Note: HB: hospital based; PB: population based; PCR-RFLP: in reaction-restriction fragment length polymorphism; NM: not mentioned.

**Table 2:** Genotype frequency distributions of lncRNA SNPs studied in included studies.

First author	Year	Gene	SNPs^a^	Type of cancer	Ethnicity	Sample size		Case			Control			Quality score	*P* ^*HWE*^	Included in meta-analysis
						Case	Control	Homozygote wild	Heterozygote	Homozygote variant	Homozygote wild	Heterozygote	Homozygote variant			
Isabel S. Carvalho	2019	CYP2R1	rs2060793 (G/A)	TC	Caucasian (Portugal)	500	500	189	236	75	183	256	61	7	**0.047**	No^c^
	2019	DHCR7	rs12785878 (T/G)	TC	Caucasian (Portugal)	500	500	150	251	99	197	234	69	7	0.971	Yes

Prajjalendra Barooah	2019	CYP2R1	rs10741657 (G/A)	HCC	Caucasian (Indian)	60	102	28	23	9	51	35	16	4.5	**0.025**	No^c^

Jianzhou Yang	2017	DHCR7	rs3829251 (G/A)	ESCC	Asian (China)	565	557	302	218	45	283	232	42	8	0.557	No^b^

Alison M. Mondul	2015	CYP2R1	rs10741657 (G/A)	BC	Caucasian (European)	8618	9960	3276	4041	1301	3708	4766	1486	6.5	0.475	Yes
	2015	DHCR7	rs12785878 (T/G)	BC	Caucasian (European)	9224	10560	4935	3620	669	5674	4052	834	5.5	**0.003**	No^c^

Tess V. Clendenen	2015	CYP2R1	rs10741657 (G/A)	BC	Caucasian (Swedish)	733	1432	200	358	175	405	720	307	7.5	0.696	Yes
	2015	DHCR7	rs12785878 (T/G)	BC	Caucasian (Swedish)	733	1433	273	371	89	571	659	203	7.5	0.562	Yes
	2015	DHCR7	rs1790349 (A/G)	BC	Caucasian (Swedish)	732	1431	348	326	58	752	571	108	7.5	0.978	Yes

Fabio Pibiri	2014	CYP2R1	rs12794714 (G/A)	CRC	African (African-American)	902	760	638	253	11	501	239	20	9.5	0.175	Yes

Touraj Mahmoudi	2014	CYP2R1	rs12794714 (G/A)	CRC	Caucasian (Iranian)	290	354	93	135	62	110	167	77	7	0.364	Yes

Wei Wang	2014	CYP2R1	rs2060793 (G/A)	BC	Mixed (Hispanic)	826	779	303	391	132	315	356	108	9.5	0.644	Yes
	2014	CYP2R1	rs2060793 (G/A)		Caucasian (non-Hispanic)	224	130	89	104	31	51	58	21	8.5	0.512	Yes
	2014	DHCR7	rs12785878 (T/G)	BC	Mixed (Hispanic)	826	779	189	410	227	185	353	241	7.5	**0.013**	No^c^
	2014	DHCR7	rs12785878 (T/G)	BC	Caucasian (non-Hispanic)	224	130	123	90	11	62	55	13	8.5	0.876	Yes
	2014	DHCR7	rs1790349 (A/G)	BC	Mixed (Hispanic)	826	779	573	227	26	527	227	25	8.5	0.927	Yes
	2014	DHCR7	rs1790349 (A/G)	BC	Caucasian (non-Hispanic)	224	130	168	52	4	95	30	5	8.5	0.195	Yes

Christian M. Lange	2013	CYP2R1	rs10741657 (G/A)	HCC	Asian (Japanese)	803	1253	320	377	106	482	597	174	8.5	0.615	Yes
	2013	DHCR7	rs12785878 (T/G)	HCC	Asian (Japanese)	803	1253	84	336	383	153	543	557	8.5	0.247	Yes
	2013	DHCR7	rs12785878 (T/G)	HCC	Caucasian (German)	116	208	63	44	9	113	77	18	8.5	0.353	Yes

Alison M. Mondul	2013	CYP2R1	rs10741657 (G/A)	PC^1^	Caucasian	9378	9986	3481	4392	1505	3789	4667	1530	9	0.137	Yes
	2013	DHCR7	rs12785878 (T/G)	PC^1^	Caucasian	9620	10225	4979	3816	825	5221	4047	957	8	2.401**E** − 05	No^c^

Laura N. Anderson	2013	CYP2R1	rs10741657 (G/A)	PC^2^	Caucasian (Canada)	625	1191	262	286	77	451	550	190	8	0.304	Yes
	2013	CYP2R1	rs12794714 (G/A)	PC^2^	Caucasian (Canada)	628	1192	180	307	141	399	559	234	8	0.131	Yes

Marissa Penna-Martinez	2012	CYP2R1	rs10741657 (G/A)	TC	Caucasian (German)	253	302	96	110	47	119	139	44	7.5	0.742	Yes
	2012	CYP2R1	rs12794714 (G/A)	TC	Caucasian (German)	253	302	78	130	45	94	144	64	7.5	0.522	Yes

Note: *P*_HWE_: the *P* value for Hardy-Weinberg equilibrium in control groups; ^a^major/minor; ^b^excluded due to the limited number for this locus; ^c^excluded due to the SNP not being in accordance with HWE. The results are in bold if *P* < 0.05; TC = thyroid cancer; HCC = hepatocellular carcinoma; ESCC = esophageal squamous cell carcinoma; BC = breast cancer; PC^1^ = prostate cancer; PC^2^ = pancreas cancer.

**Table 3:** Meta-analysis of the association between common SNPs and cancer risk.

Stratification	*N*	Heterozygote vs. wild-type			Mutation homozygote vs. wild-type			Dominant model			Recessive model			Allelic model		
		OR (95% CI)	*P*	*I* ^2^ (%)	OR (95% CI)	*P*	*I* ^2^ (%)	OR (95% CI)	*P*	*I* ^2^ (%)	OR (95% CI)	*P*	*I* ^2^(%)	OR (95% CI)	*P*	*I* ^2^ (%)
*CYP2R1*																
*rs10741657 (G/A)*	6	0.987 (0.947-1.028)	0.522	0	1.006 (0.906-1.117)	0.905	50.9	0.995 (0.957-1.034)	0.799	18.4	1.030 (0.978-1.084)	0.264	41.2	0.998 (0.949-1.050)	0.943	51.2
Ethnicity																
Caucasian	5	0.989 (0.948-1.031)	0.587	0	1.016 (0.905-1.142)	0.785	58.2	0.997 (0.959-1.038)	0.901	30.8	1.030 (0.936-1.133)	0.543	50.2	1.003 (0.948-1.061)	0.917	58.5
Asian	1	0.951 (0.786-1.152)	0.608	NA	0.918 (0.694-1.214)	0.547	NA	0.944 (0.787-1.131)	0.531	NA	0.943 (0.727-1.223)	0.658	NA	0.957 (0.840-1.089)	0.503	NA
Type of cancer																
Breast cancer	2	0.963 (0.907-1.023)	0.227	0	1.008 (0.927-1.095)	0.858	20.7	0.974 (0.920-1.031)	0.361	0	1.030 (0.955-1.111)	0.442	14.9	0.995 (0.957-1.036)	0.817	31.1
Pancreas cancer 2	1	0.895 (0.726-1.103)	0.289	NA	0.698 (0.514-0.947)	0.021	NA	0.844 (0.693-1.029)	0.093	NA	0.740 (0.557-0.984)	0.038	NA	0.848 (0.736-0.978)	0.023	NA
Prostate cancer	1	1.024 (0.963-1.090)	0.445	NA	1.071 (0.984-1.165)	0.114	NA	1.036 (0.977-1.098)	0.236	NA	1.057 (0.978-1.142)	0.165	NA	1.033 (0.992-1.076)	0.118	NA
Hepatocellular carcinoma	1	0.951 (0.786-1.152)	0.608	NA	0.918 (0.694-1.214)	0.547	NA	0.944 (0.787-1.131)	0.531	NA	0.943 (0.727-1.223)	0.658	NA	0.957 (0.840-1.089)	0.503	NA
Thyroid caner	1	0.981 (0.679-1.416)	0.918	NA	1.324 (0.810-2.164)	0.263	NA	1.063 (0.755-1.499)	0.725	NA	1.338 (0.853-2.098)	0.205	NA	1.122 (0.881-1.429)	0.352	NA
Source of controls																
HB	2	0.959 (0.903-1.018)	0.169	0	0.984 (0.905-1.070)	0.709	0	0.965 (0.912-1.021)	0.214	0	1.007 (0.933-1.088)	0.85	0	0.984 (0.946-1.024)	0.437	0
PB	4	1.012 (0.957-1.071)	0.677	0	1.020 (0.829-1.256)	0.849	64.6	1.022 (0.969-1.078)	0.415	23.5	1.032 (0.867-1.229)	0.723	60.7	1.007 (0.913-1.110)	0.89	62.3
*rs12794714 (G/A)*	4	1.007 (0.825-1.231)	0.942	51	0.908 (0.609-1.352)	0.634	68	0.993 (0.789-1.249)	0.953	66.2	0.907 (0.664-1.239)	0.539	58.9	0.968 (0.807-1.160)	0.723	72.5
Ethnicity																
Caucasian	3	1.127 (0.951-1.336)	0.167	0	1.134 (0.921-1.397)	0.236	40.9	1.130 (0.964-1.326)	0.132	10.5	1.056 (0.881-1.265)	0.558	24.8	1.074 (0.967-1.192)	0.183	41.2
African	1	0.831 (0.672-1.028)	0.088	NA	0.432 (0.205-0.910)	0.027	NA	0.800 (0.650-0.985)	0.036	NA	0.457 (0.217-0.959)	0.039	NA	0.800 (0.666-0.960)	0.017	NA
Type of cancer																
Colorectal cancer	2	0.862 (0.718-1.035)	0.111	0	0.681 (0.317-1.466)	0.326	69.1	0.841 (0.705-1.003)	0.054	0	0.717 (0.344-1.493)	0.374	68.9	**0.866 (0.753-0.997)**	**0.046**	44.1
Pancreas cancer	1	1.217 (0.973-1.524)	0.086	NA	1.336 (1.016-1.775)	0.038	NA	1.252 (1.014-1.546)	0.036	NA	1.185 (0.936-1.500)	0.157	NA	1.167 (1.017-1.339)	0.028	NA
Thyroid caner	1	1.088 (0.742-1.595)	0.666	NA	0.847 (0.522-1.377)	0.504	NA	1.014 (0.706-1.455)	0.94	NA	0.805 (0.526-1.230)	0.315	NA	0.939 (0.740-1.191)	0.604	NA
Source of controls																
HB	1	0.956 (0.669-1.367)	0.806	NA	0.952 (0.617-1.469)	0.825	NA	0.955 (0.684-1.333)	0.787	NA	0.978 (0.671-1.427)	0.909	NA	0.973 (0.780-1.213)	0.806	NA
PB	3	1.023 (0.787-1.331)	0.864	66.9	0.856 (0.476-1.538)	0.602	78	1.004 (0.742-1.360)	0.978	77.3	0.839 (0.522-1.348)	0.468	72.5	0.963 (0.754-1.231)	0.765	81.6
*rs2060793 (G/A)*	2	1.121 (0.923-1.362)	0.247	0	1.184 (0.902-1.554)	0.223	19	1.136 (0.946-1.364)	0.172	0	1.113 (0.866-1.430)	0.402	6.3	1.098 (0.964-1.250)	0.16	8.4
*DHCR7*																
*rs12785878 (T/G)*	*5*	**1.168 (1.027-1.328)**	**0.018**	8.3	1.074 (0.736-1.569)	0.71	73.1	1.136 (0.935-1.381)	0.2	53.2	1.017 (0.762-1.357)	0.91	67.5	1.064 (0.906-1.250)	0.448	70.1
Ethnicity																
Caucasian	4	**1.178 (1.021-1.358)**	**0.024**	30.1	0.980 (0.562-1.710)	0.944	79.2	1.108 (0.854-1.436)	0.441	64.8	0.929 (0.584-1.477)	0.756	73.7	1.031 (0.814-1.305)	0.802	77.2
Asian	1	1.127 (0.836-1.520)	0.433	NA	1.252 (0.931-1.684)	0.136	NA	1.191 (0.898-1.579)	0.226	NA	1.139 (0.954-1.361)	0.15	NA	1.120 (0.980-1.281)	0.097	NA
Type of cancer																
Breast cancer	2	1.048 (0.756-1.454)	0.778	50.1	0.699 (0.341-1.433)	0.328	63.5	0.961 (0.658-1.404)	0.838	63.9	0.795 (0.616-1.025)	0.077	42.4	0.899 (0.665-1.215)	0.488	66.1
Hepatocellular carcinoma	2	1.098 (0.851-1.415)	0.472	0	1.209 (0.913-1.599)	0.185	0	1.135 (0.893-1.442)	0.302	0	1.127 (0.947-1.341)	0.177	0	1.102 (0.972-1.250)	0.129	0
Thyroid caner	1	1.409 (1.068-1.859)	0.015	NA	1.884 (1.297-2.738)	0.001	NA	1.517 (1.167-1.972)	0.002	NA	1.542 (1.102-2.158)	0.012	NA	1.376 (1.150-1.645)	<0.001	NA
Source of controls																
HB	2	1.098 (0.852-1.415)	0.471	0	1.209 (0.913-1.599)	0.185	0	1.135 (0.893-1.442)	0.302	0	1.127 (0.947-1.341)	0.177	0	1.102 (0.972-1.250)	0.129	0
PB	3	**1.193 (1.028-1.385)**	**0.02**	49.3	0.988 (0.501-1.945)	0.971	85.9	1.125 (0.815-1.552)	0.474	75.3	0.925 (0.528-1.623)	0.787	82.3	1.038 (0.777-1.388)	0.8	84.5
*rs1790349 (A/G)*	3	1.060 (0.850-1.323)	0.605	52	1.056 (0.793-1.407)	0.71	0	1.043 (0.837-1.300)	0.705	55.1	0.998 (0.754-1.319)	0.986	0	1.048 (0.942-1.167)	0.391	45.7
Ethnicity																
Caucasian	2	**1.201 (1.008-1.431)**	**0.04**	0	1.094 (0.784-1.526)	0.598	44	1.180 (0.998-1.396)	0.053	21.2	1.003 (0.727-1.386)	0.983	30.6	1.110 (0.972-1.266)	0.122	37.4
Mixed	1	0.920 (0.739-1.145)	0.453	NA	0.957 (0.545-1.677)	0.877	NA	0.923 (0.748-1.140)	0.458	NA	0.980 (0.561-1.712)	0.944	NA	0.940 (0.783-1.128)	0.505	NA

Note: OR: odds ratio; CI: confidence interval. The results are in bold if *P* < 0.05.

**Table 4:** The results of Begg's and Egger's test for the publication bias.

Comparison type	Begg's test		Egger's test	
	*Z* value	*P* value	*t* value	*P* value
CYP2R1 rs10741657 (G/A)				
Heterozygote vs. homozygote wild	0	1	-0.7	0.521
Homozygote variant vs. homozygote wild	0.38	0.707	-0.73	0.503
Dominant model	0	1	-0.53	0.627
Recessive model	0	1	-0.29	0.787
Allelic model	0.75	0.452	-0.38	0.722
CYP2R1 rs12794714 (G/A)				
Heterozygote vs. homozygote wild	0.34	0.734	0.21	0.851
Homozygote variant vs. homozygote wild	1.02	0.308	-2.84	0.105
Dominant model	0.34	0.734	-0.01	0.994
Recessive model	1.7	**0.089**	-9.45	**0.011**
Allelic model	0.34	0.734	-1.12	0.38
CYP2R1 rs2060793 (G/A)				
Heterozygote vs. homozygote wild	0	1	NA	NA
Homozygote variant vs. homozygote wild	0	1	NA	NA
Dominant model	0	1	NA	NA
Recessive model	0	1	NA	NA
Allelic model	0	1	NA	NA
DHCR7 rs12785878 (T/G)				
Heterozygote vs. homozygote wild	0.24	0.806	-1.64	0.2
Homozygote variant vs. homozygote wild	-0.24	1	-1.76	0.177
Dominant model	-0.24	1	-1.74	0.18
Recessive model	0.24	0.806	-1.15	0.332
Allelic model	0.24	0.806	-1.56	0.217
DHCR7 rs1790349 (A/G)				
Heterozygote vs. homozygote wild	0	1	0.18	0.884
Homozygote variant vs. homozygote wild	0	1	0.13	0.92
Dominant model	0	1	-0.44	0.737
Recessive model	0	1	-2.34	0.257
Allelic model	1.04	0.296	-0.9	0.532

Note: the results are in bold if *P* < 0.1.

**Table 5:** False-positive report probability values for correlations between genotype frequency of DHCR7 and CYP2R1 and cancer risk.

Genotype	OR (95% CI)	*P* value	Statistical power^a^	Prior probability^b^				
				0.25	0.1	0.01	0.001	0.0001
*rs12785878 (T/G)*								
GT vs. TT (overall)	1.168 (1.027-1.328)	0.018	0.312	*0.235*	*0.390*	0.853	0.983	0.998
GT vs. TT (Caucasian)	1.178 (1.021-1.358)	0.024	0.271	*0.321*	*0.496*	0.899	0.989	0.999
GT vs. TT (PB)	1.193 (1.028-1.385)	0.02	0.264	*0.288*	*0.457*	0.885	0.986	0.999
*rs1790349 (A/G)*								
GA vs. AA (Caucasian)	1.201 (1.008-1.431)	0.04	0.290	*0.424*	0.605	0.933	0.993	0.999
*rs12794714 (G/A)*								
AA vs. GG (CRC)	0.866 (0.753-0.997)	0.046	0.367	*0.401*	0.582	0.927	0.992	0.999

Note: CI: confidence interval; OR: odds ratio; ^a^statistical power was computed using the sample size of case and control, OR, and *P* values; ^b^the false-positive report probability is in italics if the value < 0.5.

## Data Availability

The authors declare that all relevant data are presented within the paper.

## Abbreviations

SNP: Single nucleotide polymorphism
GWAS: Genome-wide association studies
ORs: Odds ratios
CI: Confidence intervals
HWE: Hardy-Weinberg equilibrium
FPRP: False-positive report probability
ESE: Exon splicing enhancer
ESS: Exon splicing silencer.

## Supplementary Materials

Supplementary Materials Table S1: ORs (95% CIs) of sensitivity analysis.

## References

[1] Jiang X., O’Reilly P. F., Aschard H. (2018). Genome-wide association study in 79,366 European-ancestry individuals informs the genetic architecture of 25-hydroxyvitamin D levels. *Nature Communications*.

[2] Martineau A. R., Jolliffe D. A., Hooper R. L. (2017). Vitamin D supplementation to prevent acute respiratory tract infections: systematic review and meta-analysis of individual participant data. *BMJ*.

[3] Pilz S., Verheyen N., Grubler M. R., Tomaschitz A., Marz W. (2016). Vitamin D and cardiovascular disease prevention. *Nature Reviews. Cardiology*.

[4] Berardi S., Giardullo L., Corrado A., Cantatore F. P. (2020). Vitamin D and connective tissue diseases. *Inflammation Research*.

[5] Fernandes de Abreu D. A., Eyles D., Feron F. (2009). Vitamin D, a neuro-immunomodulator: implications for neurodegenerative and autoimmune diseases. *Psychoneuroendocrinology*.

[6] Wu X., Hu W., Lu L. (2019). Repurposing vitamin D for treatment of human malignancies via targeting tumor microenvironment. *Acta Pharmaceutica Sinica B*.

[7] Huang Z., Zhang Y., Li H. (2019). Vitamin D promotes the cisplatin sensitivity of oral squamous cell carcinoma by inhibiting LCN2-modulated NF-*κ*B pathway activation through RPS3. *Cell Death & Disease*.

[8] Song D., Deng Y., Liu K. (2019). Vitamin D intake, blood vitamin D levels, and the risk of breast cancer: a dose-response meta-analysis of observational studies. *Aging (Albany NY)*.

[9] Mahmoudi T., Karimi K., Arkani M. (2014). Lack of associations between vitamin D metabolism-related gene variants and risk of colorectal cancer. *Asian Pacific journal of cancer prevention: APJCP*.

[10] Shui I. M., Mucci L. A., Kraft P. (2012). Vitamin D-related genetic variation, plasma vitamin D, and risk of lethal prostate cancer: a prospective nested case-control study. *Journal of the National Cancer Institute*.

[11] Anderson L. N., Cotterchio M., Knight J. A., Borgida A., Gallinger S., Cleary S. P. (2013). Genetic variants in vitamin d pathway genes and risk of pancreas cancer; results from a population-based case-control study in Ontario, Canada. *PloS one*.

[12] Carvalho I. S., Goncalves C. I., Almeida J. T. (2019). Association of vitamin D pathway genetic variation and thyroid cancer. *Genes*.

[13] Barooah P., Saikia S., Bharadwaj R. (2019). Role of VDR, GC, and CYP2R1 polymorphisms in the development of hepatocellular carcinoma in hepatitis C virus-infected patients. *Genetic Testing and Molecular Biomarkers*.

[14] Dovnik A., Fokter Dovnik N. (2020). Vitamin D and ovarian cancer: systematic review of the literature with a focus on molecular mechanisms. *Cell*.

[15] Zhang Y., Fang F., Tang J. (2019). Association between vitamin D supplementation and mortality: systematic review and meta-analysis. *BMJ*.

[16] Wang T. J., Zhang F., Richards J. B. (2010). Common genetic determinants of vitamin D insufficiency: a genome-wide association study. *Lancet*.

[17] Afshan F. U., Masood A., Nissar B. (2021). Promoter hypermethylation regulates vitamin D receptor (VDR) expression in colorectal cancer-a study from Kashmir valley. *Cancer Genetics*.

[18] Gnagnarella P., Raimondi S., Aristarco V. (2021). Ethnicity as modifier of risk for vitamin D receptors polymorphisms: comprehensive meta-analysis of all cancer sites. *Critical Reviews in Oncology/Hematology*.

[19] Latacz M., Snarska J., Kostyra E., Fiedorowicz E., Savelkoul H. F., Grzybowski R. (2020). Cieslinska A: Single nucleotide polymorphisms in 25-hydroxyvitamin D3 1-alpha-hydroxylase (CYP27B1) gene: the risk of malignant tumors and other chronic diseases. *Nutrients*.

[20] O'Brien K. M., Sandler D. P., Kinyamu H. K., Taylor J. A., Weinberg C. R. (2017). Single-nucleotide polymorphisms in vitamin D-related genes may modify vitamin D-breast cancer associations. *Cancer Epidemiology, Biomarkers & Prevention: A Publication of the American Association for Cancer Research, cosponsored by the American Society of Preventive Oncology*.

[21] Voutsadakis I. A. (2020). Vitamin D receptor (VDR) and metabolizing enzymes CYP27B1 and CYP24A1 in breast cancer. *Molecular Biology Reports*.

[22] Arem H., Yu K., Xiong X. (2015). Vitamin D metabolic pathway genes and pancreatic cancer risk. *PLoS One*.

[23] La Torre G., Chiaradia G., Gianfagna F., Laurentis A., Boccia S., Ricciardi W. (2006). Quality assessment in meta-analysis. *Italian Journal of Public Health*.

[24] Lv Z., Xu Q., Yuan Y. (2017). A systematic review and meta-analysis of the association between long non- coding RNA polymorphisms and cancer risk. *Mutation Research*.

[25] Wacholder S., Chanock S., Garcia-Closas M., El Ghormli L., Rothman N. (2004). Assessing the probability that a positive report is false: an approach for molecular epidemiology studies. *Journal of the National Cancer Institute*.

[26] Strawbridge R. J., Deleskog A., McLeod O. (2014). A serum 25-hydroxyvitamin D concentration-associated genetic variant in DHCR7 interacts with type 2 diabetes status to influence subclinical atherosclerosis (measured by carotid intima-media thickness). *Diabetologia*.

[27] Lu L., Sheng H., Li H. (2012). Associations between common variants in GC and DHCR7/NADSYN1 and vitamin D concentration in Chinese Hans. *Human Genetics*.

[28] Ahn J., Yu K., Stolzenberg-Solomon R. (2010). Genome-wide association study of circulating vitamin D levels. *Human Molecular Genetics*.

[29] Sunkar S., Neeharika D. (2020). CYP2R1 and CYP27A1 genes: an in silico approach to identify the deleterious mutations, impact on structure and their differential expression in disease conditions. *Genomics*.

[30] Thacher T. D., Fischer P. R., Singh R. J., Roizen J., Levine M. A. (2015). CYP2R1 mutations impair generation of 25-hydroxyvitamin D and cause an atypical form of vitamin D deficiency. *The Journal of Clinical Endocrinology and Metabolism*.

[31] Kwak J. H., Paik J. K. (2020). Vitamin D status and gastric cancer: a cross-sectional study in Koreans. *Nutrients*.

[32] Hu M. J., Niu Q. S., Wu H. B. (2020). Association of thyroid cancer risk with plasma 25-hydroxyvitamin D and vitamin D binding protein: a case-control study in China. *Journal of Endocrinological Investigation*.

[33] Gao J., Wei W., Wang G., Zhou H., Fu Y., Liu N. (2018). Circulating vitamin D concentration and risk of prostate cancer: a dose-response meta-analysis of prospective studies. *Therapeutics and Clinical Risk Management*.

[34] Zhang L., Zou H., Zhao Y. (2019). Association between blood circulating vitamin D and colorectal cancer risk in Asian countries: a systematic review and dose-response meta-analysis. *BMJ Open*.

[35] Yang J., Wang H., Ji A. (2017). Vitamin D signaling pathways confer the susceptibility of esophageal squamous cell carcinoma in a northern Chinese population. *Nutrition and Cancer*.

[36] Mondul A. M., Shui I. M., Yu K. (2015). Vitamin D-associated genetic variation and risk of breast cancer in the breast and prostate cancer cohort consortium (BPC3). *Cancer Epidemiology, Biomarkers & Prevention: A Publication of the American Association for Cancer Research, cosponsored by the American Society of Preventive Oncology*.

[37] Clendenen T. V., Ge W., Koenig K. L. (2015). Genetic polymorphisms in vitamin D metabolism and signaling genes and risk of breast cancer: a nested case-control study. *PLoS One*.

[38] Pibiri F., Kittles R. A., Sandler R. S. (2014). Genetic variation in vitamin D-related genes and risk of colorectal cancer in African Americans. *Cancer Causes & Control*.

[39] Wang W., Ingles S. A., Torres-Mejía G. (2014). Genetic variants and non-genetic factors predict circulating vitamin D levels in Hispanic and non-Hispanic white women: the Breast Cancer Health Disparities Study. *International Journal of Molecular Epidemiology and Genetics*.

[40] Lange C. M., Miki D., Ochi H. (2013). Genetic analyses reveal a role for vitamin D insufficiency in HCV-associated hepatocellular carcinoma development. *PLoS One*.

[41] Mondul A. M., Shui I. M., Yu K. (2013). Genetic variation in the vitamin d pathway in relation to risk of prostate cancer--results from the breast and prostate cancer cohort consortium. *Cancer Epidemiology, Biomarkers & Prevention: A Publication of the American Association for Cancer Research, cosponsored by the American Society of Preventive Oncology*.

[42] Penna-Martinez M., Ramos-Lopez E., Stern J. (2012). Impaired vitamin D activation and association with CYP24A1 haplotypes in differentiated thyroid carcinoma. *Thyroid*.

