# Supplementary material for: Association of Polymorphisms in Vitamin D-Metabolizing Enzymes DHCR7 and CYP2R1 with Cancer Susceptibility: A Systematic Review and Meta-Analysis
Source: Dis Markers. 2021 May 22;2021:6615001. doi: 10.1155/2021/6615001 (PMC8164542; doi:10.1155/2021/6615001)
Supplement: Supplementary Materials — Table S1: ORs (95% CIs) of sensitivity analysis. [file 6615001.f1.DOCX]

| Table S1. ORs(95% CI) of sensitivity analysis | | | | | |
| --- | --- | --- | --- | --- | --- |
| Excluding literature one by one | Heterozygote vs. wild-type | Mutation homozygote vs. wild-type | Dominant model | Recessive model | Allelic model |
|  | OR (95%CI) | OR (95%CI) | OR (95%CI) | OR (95%CI) | OR (95%CI) |
| CYP2R1 rs10741657 (G/A） |  |  |  |  |  |
| Overall | 0.987(0.947-1.028) | 1.006(0.906-1.117) | 0.995(0.957-1.034) | 1.030(0.978-1.084) | 0.998(0.949-1.050) |
| Alison M. Mondul | 1.007(0.954-1.063) | 1.002(0.847-1.184) | 1.016(0.965-1.069) | 1.041(0.973-1.113) | 0.998(0.923-1.079) |
| TessV.Clendenen | 0.986(0.946-1.028) | 0.985(0.877-1.107) | 0.993(0.954-1.033) | 1.023(0.970-1.079) | 0.988(0.935-1.044) |
| Christian M. Lange | 0.988(0.948-1.031) | 1.016(0.905-1.142) | 0.997(0.959-1.038) | 1.034(0.981-1.090) | 1.003(0.948-1.061) |
| AlisonM.Mondul | 0.958(0.907-1.012) | 0.975(0.835-1.139) | 0.964(0.915-1.015) | 1.009(0.942-1.081) | 0.982(0.916-1.053) |
| Marissa Penna-Martinez | 0.987(0.947-1.028) | 0.994(0.893-1.107) | 0.994(0.956-1.034) | 1.026(0.974-1.081) | 0.993(0.942-1.046) |
| Laura N. Anderson | 0.991(0.950-1.033) | 1.036(0.978-1.098) | 1.001(0.963-1.042) | 1.042(0.989-1.098) | 1.013(0.981-1.046) |
| CYP2R1 rs12794714 (G/A) |  |  |  |  |  |
| Overall | 1.007(0.825-1.231) | 0.908(0.609-1.352) | 0.993(0.789-1.249) | 0.907(0.664-1.239) | 0.968(0.807-1.160) |
| Fabio Pibiri | 1.127(0.951-1.336) | 1.084(0.811-1.448) | 1.121(0.943-1.332) | 1.034(0.831-1.288) | 1.049(0.907-1.213) |
| Touraj Mahmoudi1 | 1.023(0.787-1.331) | 0.856(0.476-1.538) | 1.004(0.742-1.360) | 0.839(0.522-1.348) | 0.963(0.754-1.231) |
| Marissa Penna-Martinez | 0.991(0.768-1.278) | 0.903(0.529-1.541) | 0.988(0.734-1.328) | 0.922(0.615-1.383) | 0.974(0.769-1.234) |
| Laura N. Anderson | 0.900(0.763-1.061) | 0.772(0.520-1.146) | 0.872(0.744-1.021) | 0.791(0.556-1.126) | 0.885(0.782-1.003) |
| CYP2R1 rs2060793 (G/A) |  |  |  |  |  |
| Overall | 1.121(0.923-1.362) | 1.184(0.902-1.554) | 1.136(0.946-1.364) | 1.113(0.866-1.430) | 1.098(0.964-1.250) |
| Wei Wang | 1.028(0.642-1.645) | 0.846(0.441-1.624) | 0.979(0.629-1.524) | 0.834(0.457-1.522) | 0.942(0.687-1.290) |
| Wei Wang | 1.142(0.923-1.413) | 1.271(0.942-1.714) | 1.172(0.958-1.433) | 1.182(0.897-1.557) | 1.132(0.982-1.306) |
| DHCR7 rs12785878 (T/G) |  |  |  |  |  |
| Overall | 1.168(1.027-1.328) | 1.074(0.736-1.569) | 1.136(0.935-1.381) | 1.017(0.762-1.357) | 1.064(0.906-1.250) |
| Isabel S. Carvalho | 1.110(0.960-1.283) | 0.938(0.667-1.317) | 1.069(0.915-1.249) | 0.907(0.674-1.220) | 1.007(0.880-1.152) |
| TessV.Clendenen | 1.161(0.976-1.380) | 1.109(0.678-1.815) | 1.123(0.844-1.494) | 1.086(0.766-1.541) | 1.069(0.856-1.334) |
| Wei Wang | 1.204(1.053-1.377) | 1.222(0.867-1.723) | 1.212(1.032-1.424) | 1.099(0.844-1.431) | 1.126(0.976-1.299) |
| Christian M. Lange | 1.178(1.021-1.358) | 0.980(0.562-1.710) | 1.108(0.854-1.436) | 0.929(0.584-1.477) | 1.031(0.814-1.305) |
| Christian M. Lange | 1.180(1.032-1.348) | 1.094(0.715-1.674) | 1.153(0.920-1.443) | 1.025(0.745-1.411) | 1.074(0.896-1.287) |
| DHCR7 rs1790349 (A/G) |  |  |  |  |  |
| Overall | 1.060(0.850-1.323) | 1.056(0.793-1.407) | 1.043(0.837-1.300) | 0.998(0.754-1.319) | 1.048(0.942-1.167) |
| TessV.Clendenen | 0.929(0.759-1.136) | 0.856(0.511-1.435) | 0.920(0.758-1.117) | 0.875(0.524-1.462) | 0.926(0.782-1.096) |
| Wei Wang | 1.201(1.008-1.431) | 1.094(0.784-1.526) | 1.152(0.911-1.456) | 1.003(0.727-1.386) | 1.110(0.972-1.266) |
| Wei Wang | 1.071(0.804-1.429) | 1.100(0.820-1.476) | 1.069(0.812-1.406) | 1.034(0.777-1.376) | 1.062(0.951-1.186) |
| Note: OR, odds ratio; CI, confidence interval. | | | | | |
